# Work-related well-being among hepatobiliary surgical nurses: a structural equation modeling study

**DOI:** 10.3389/fpubh.2026.1772238

**Published:** 2026-02-12

**Authors:** Yu Wang, Lina Zhou, Yun Guo

**Affiliations:** Department of Hepatobiliary Surgery, The First Affiliated Hospital with Nanjing Medical University, Nanjing, Jiangsu, China

**Keywords:** hepatobiliary surgery nurse, professional benefits, psychological resilience, work happiness, work stressors

## Abstract

**Background:**

Hepatobiliary surgical nurses face high workloads, complex care demands, and continuous psychological strain, which may impair their work-related well-being. Psychological resilience, occupational stress, and perceived professional benefits are key psychological resources and risks, yet their combined effects in this population are not well understood.

**Methods:**

In this cross-sectional study, 280 hepatobiliary surgical nurses from eight hospitals in Jiangsu Province, China, were recruited by convenience sampling. Psychological resilience, occupational stress, perceived professional benefits, and work-related well-being were assessed using validated Chinese versions of the Connor–Davidson Resilience Scale, Chinese Nurses Stressor Scale, Nurses' Perceived Professional Benefits Scale, and Nurse Work Happiness Scale. Data were analyzed using descriptive statistics, Pearson correlations, multiple linear regression, and structural equation modeling.

**Results:**

Nurses showed lower psychological resilience than the national norm and moderate perceived professional benefits. Occupational stress mainly stemmed from patient care, whereas work-related well-being was most strongly related to perceived work value. Regression analyses identified psychological resilience, occupational stress, perceived professional benefits, number of children, employment type, and monthly income as significant predictors of work-related well-being (all *p* < 0.05). The structural equation model demonstrated good fit and indicated that resilience and professional benefits had direct positive effects on well-being, occupational stress had a direct negative effect, and resilience and professional benefits partially mediated the impact of stress on work-related well-being.

**Conclusion:**

Work-related well-being among hepatobiliary surgical nurses is shaped by both occupational stress and internal psychological resources. Interventions that reduce stress and strengthen nurses' resilience and perceived professional benefits may be effective strategies to improve well-being in hepatobiliary surgery settings.

## 1 Introduction

Well-being is generally defined as an individual's positive emotional state and cognitive appraisal of life. When applied to the workplace, work-related well-being refers to employees' subjective positive emotions and evaluations toward their professional role (1). For nurses, higher levels of work-related well-being can reduce burnout, promote workforce stability, and enhance patient satisfaction (2–4). Therefore, its quantitative assessment and optimization have important implications.

Hepatobiliary surgery units are characterized by high clinical acuity, heavy workloads, complex perioperative management, and a high incidence of complications. Nurses working in these settings frequently encounter critically ill patients, unpredictable disease progression, and demanding technical procedures, which can lead to sustained occupational stress and emotional exhaustion (5). In such high-risk environments, maintaining nurses' well-being is crucial not only for their physical and mental health, but also for ensuring the safety and quality of patient care.

Work-related well-being is shaped by multiple factors, including personal characteristics, workplace conditions, and social support. Previous research has often focused on external determinants while overlooking the role of internal psychological resources (6). Psychological resilience, an essential internal resource, is defined as the ability to adaptively regulate psychological and behavioral responses when facing environmental challenges (7, 8). It may therefore serve as a key factor influencing well-being. Similarly, occupational stress exerts dual effects: it can foster growth but may also impair efficiency and compromise nurses' physical and mental health (9–11). Exploring its relationship with well-being is thus crucial. Another relevant construct is professional benefit perception, which describes the positive emotional state derived from recognizing the value of one's profession (12, 13). This perception strengthens professional identity and retention intention, and may further influence nurses' well-being.

Previous studies have consistently shown that psychological resilience plays a protective role in nurses' occupational well-being, particularly in high-stress clinical environments. Research among mental health nurses and other frontline nursing groups has demonstrated that higher levels of resilience are associated with better professional quality of life, lower emotional exhaustion, and improved psychological well-being (14, 15). Resilience enables nurses to adapt to ongoing workplace stressors, regulate emotional responses, and maintain a sense of professional efficacy under pressure.

Occupational stress, by contrast, has been widely recognized as a major risk factor for impaired well-being among nurses. Systematic reviews and empirical studies indicate that excessive workload, emotional labor, and responsibility for critically ill patients are strongly associated with reduced job satisfaction, increased burnout, and poorer mental health outcomes (16, 17). In high-intensity specialties, such as mental health and surgical nursing, sustained exposure to occupational stress may erode nurses' psychological resources if adequate buffering mechanisms are not present. Emerging evidence further suggests that positive psychological and professional resources can mitigate the negative impact of occupational stress. Perceived professional benefits, reflecting nurses' recognition of professional value, personal growth, and social affirmation, have been linked to stronger professional identity and higher work-related well-being (18, 19). Together, these findings highlight the importance of examining both occupational stressors and protective psychological resources when investigating work-related well-being among nurses.

The present study was theoretically grounded in the Job Demands–Resources (JD-R) model, which posits that employee well-being is determined by the balance between job demands and available personal and job-related resources (20, 21). According to this framework, excessive job demands, such as occupational stress, may undermine well-being, whereas personal resources (e.g., psychological resilience) and job-related resources (e.g., perceived professional benefits) can buffer the negative effects of demands and promote positive work-related outcomes. In the context of nursing, particularly in high-intensity specialties, the JD-R model has been widely applied to explain variations in occupational well-being, burnout, and mental health. Based on this model, the present study conceptualized occupational stress as a job demand, psychological resilience as a key personal resource, and perceived professional benefits as a positive job-related resource, with work-related well-being as the primary outcome.

To date, few studies have simultaneously examined psychological resilience, occupational stress, perceived professional benefits, and work-related well-being among hepatobiliary surgical nurses using structural equation modeling. The mechanisms through which these variables interact, particularly the potential mediating roles of resilience and professional benefit perception, remain unclear. Therefore, this study targeted hepatobiliary surgical nurses in Jiangsu Province and aimed to: (1) describe the status of psychological resilience, occupational stress, perceived professional benefits, and work-related well-being; (2) identify the main influencing factors of work-related well-being; and (3) construct and test a structural equation model to clarify the pathways linking resilience, occupational stress, perceived professional benefits, and work-related well-being. The findings are expected to provide an empirical basis for nursing managers to develop targeted interventions to enhance nurses' well-being and promote team stability in hepatobiliary surgery units.

## 2 Methods

### 2.1 Study design and setting

This was a cross-sectional, questionnaire-based study conducted among hepatobiliary surgical nurses from eight general hospitals in Jiangsu Province, China, between 01/05/2024 and 31/08/2024. The study was reviewed and approved by the Ethics Committee of Nanjing Medical University. Approval was granted on 26/04/2024. All procedures complied with the Declaration of Helsinki. Participants were prospectively recruited and provided written informed consent before completing the questionnaire. The study was approved by the Ethics Committee of Nanjing Medical University (Approval No. 2024-237). Written informed consent was obtained from all participants prior to data collection.

### 2.2 Participants and sampling

Registered nurses working in hepatobiliary surgery departments were recruited using convenience sampling. Inclusion criteria were as follows: (1) provided written informed consent and voluntarily participated in the study; (2) held a valid Registered Nurse license in the People's Republic of China and were currently engaged in clinical nursing in a hepatobiliary surgery unit; (3) were in good physical condition and able to perform routine nursing duties. Exclusion criteria were: (1) refusal to participate; (2) being on maternity leave, sick leave, or other types of leave during the survey period; (3) participation in short-term rotation, advanced training, or further education away from the hepatobiliary unit.

A total of 280 nurses completed valid questionnaires and were included in the final analysis.

### 2.3 Sample size calculation

For multiple linear regression, the required sample size is generally 10–20 times the number of independent variables. In this study, 11 demographic variables were included, yielding a required sample size of 110–220. In addition, according to recommendations for structural equation modeling, 5–10 participants per observed variable were added; with four core study variables, an additional 20–40 cases were required. To account for potential missing data and invalid responses, the estimated sample size was further increased by 20%. Thus, the target sample size ranged from 156 to 312. The final sample of 280 nurses met these requirements.

### 2.4 Measures

All participants completed a general information questionnaire and four standardized scales.

### 2.5 General information questionnaire

This self-designed questionnaire collected demographic and work-related characteristics, including gender, age, marital status, number of children, employment type, years of experience in hepatobiliary surgery, highest educational level, professional title, administrative position, monthly income, and place of birth. The selection of demographic and work-related variables was informed by prior nursing well-being research and the Job Demands–Resources framework, which highlights the role of socioeconomic and employment characteristics in shaping occupational well-being.

### 2.6 Psychological resilience

Psychological resilience was assessed using the 25-item Connor–Davidson Resilience Scale (CD-RISC), developed by Connor and Davidson and adapted for Chinese populations by Xiao and Zhang. The scale comprises three dimensions: tenacity, strength, and optimism. Each item is rated on a 5-point Likert scale from 0 (“never”) to 4 (“almost always”), with total scores ranging from 0 to 100. Higher scores indicate higher levels of psychological resilience. In the present study, the CD-RISC demonstrated good internal consistency, with a Cronbach's alpha coefficient of 0.91.

### 2.7 Occupational stress

Occupational stress was measured by the Chinese Nurses' Stressor Scale (CNSS), developed by Li and Liu based on Gray–Toft's nurse stress framework. The scale consists of 35 items across five dimensions: professional and work-related issues, workload and time allocation, work environment and equipment, patient care, and management and interpersonal relationships. Items are rated on a 4-point Likert scale from 1 (“none”) to 4 (“very high”), with higher scores indicating greater perceived occupational stress. In the present study, the CNSS showed excellent internal consistency, with a Cronbach's alpha coefficient of 0.98.

### 2.8 Perceived professional benefits

Perceived professional benefits were evaluated using the Nurses' Perceived Professional Benefits Scale (NPPB), developed by Hu. The scale contains 33 items in five dimensions: recognition by relatives and friends, sense of team belonging, positive career perception, harmonious nurse–patient relationship, and personal growth. Each item is scored on a 5-point Likert scale from 1 (“strongly disagree”) to 5 (“strongly agree”), with higher scores reflecting stronger perceived professional benefits. In the present study, the NPPB demonstrated excellent internal consistency, with a Cronbach's alpha coefficient of 0.958.

### 2.9 Work-related well-being

Work-related well-being was assessed using the Nurse Work Happiness Scale (NEHS), developed by Chen and Liu for clinical nurses in China. The NEHS comprises 19 items across five domains: welfare and benefits, interpersonal relationships, work value, management, and job characteristics. Items are rated on a 6-point Likert scale from 1 (“strongly disagree”) to 6 (“strongly agree”). Higher scores indicate higher levels of work-related well-being. In the present study, the NEHS showed good internal consistency, with a Cronbach's alpha coefficient of 0.914.

Confirmatory factor analysis was not conducted in the present study, as all instruments were established Chinese versions with stable factor structures that have been validated in previous large-sample studies.

### 2.10 Data collection procedures

Data were collected from May to August 2024. After obtaining approval from hospital nursing departments, investigators explained the purpose, content, and procedures of the study to head nurses of hepatobiliary surgery units. With their support, eligible nurses were invited to participate and completed the questionnaires anonymously, either in paper form or via a secure online survey platform. Unified instructions were provided, and trained research assistants checked each questionnaire for completeness and logical consistency. Questionnaires with missing key information or obviously invalid responses were excluded.

### 2.11 Statistical analysis

Data were analyzed using IBM SPSS Statistics version 29.0. Descriptive statistics (mean, standard deviation, frequency, and percentage) were used to summarize demographic characteristics and scale scores. Independent-samples *t* tests and one-way analysis of variance (ANOVA) were applied to compare differences in psychological resilience, occupational stress, perceived professional benefits, and work-related well-being across demographic groups. Pearson correlation analysis was performed to examine the relationships among the four main variables. Multiple linear regression analysis was used to identify factors associated with work-related well-being. Before multiple linear regression analysis, multicollinearity among independent variables was assessed using variance inflation factors (VIF). A VIF value < 5 was considered acceptable, indicating no serious multicollinearity. To adhere to the principle of model parsimony and reduce the risk of overfitting, a sensitivity analysis was conducted by refitting the regression models after excluding non-significant covariates, and the results of the main variables were compared between the full models and the parsimonious models. Structural equation modeling (SEM) was further employed to explore the pathways linking psychological resilience, occupational stress, perceived professional benefits, and work-related well-being. Model fit was evaluated using commonly recommended indices, including the goodness-of-fit index (GFI), root mean square error of approximation (RMSEA), root mean square residual (RMR), comparative fit index (CFI), normed fit index (NFI), and non-normed fit index (NNFI). A two-tailed *p* value < 0.05 was considered statistically significant.

## 3 Results

### 3.1 Demographic characteristics

A total of 280 hepatobiliary surgical nurses completed valid questionnaires. Their demographic and work-related characteristics are summarized in Table 1. The majority were female (93.21%), and the largest age group was 26–40 years (40.00%). Most participants were married (71.43%), and nurses with one child accounted for the highest proportion (41.43%). Only 25.00% held permanent positions, whereas 43.93% were employed as agency personnel and 31.07% as contract nurses. The most common length of hepatobiliary surgical experience was 6–10 years (35.00%). Regarding education, 69.29% held a bachelor's degree. The most frequent professional title was nurse practitioner (41.07%), and 95.71% had no administrative position. Monthly income was relatively evenly distributed, with the largest group earning 5,000–8,000 RMB (41.07%). In terms of place of birth, 65.36% were from rural areas and 34.64% from urban areas (Table 1).

**Table 1:** Demographic characteristics of hepatobiliary surgical nurses.

**Name**	**Option**	**Frequency**	**Percentage (%)**	**Cumulative percentage (%)**
Gender	Male	19	6.79	6.79
	Female	261	93.21	100.00
Age (years)	≤ 25	26	9.29	9.29
	26–40	112	40.00	49.29
	41–55	78	27.86	77.14
	≥55	64	22.86	100.00
Marital status	Unmarried	42	15.00	15.00
	Married	200	71.43	86.43
	Divorced	38	13.57	100.00
	Widowed	100	35.71	35.71
Number of children	1	116	41.43	77.14
	≥2	64	22.86	100.00
Employment type	Permanent	70	25.00	25.00
	Agency	123	43.93	68.93
	Contract	87	31.07	100.00
Years in hepatobiliary surgery	≤ 5	59	21.07	21.07
	6–10	98	35.00	56.07
	11–15	49	17.50	73.57
	16–20	63	22.50	96.07
	≥21	11	3.93	100.00
Education level	Secondary vocational school	31	11.07	11.07
	Junior college	43	15.36	26.43
	Bachelor's degree	194	69.29	95.71
	Master's degree or above	12	4.29	100.00
Professional title	Registered Nurse	54	19.29	19.29
	Senior Nurse	115	41.07	60.36
Administrative position	Supervising Nurse	95	33.93	94.29
	Associate Chief Nurse	8	2.86	97.14
	Chief Nurse	8	2.86	100.00
	Director of Nursing	3	1.07	1.07
	Nurse Manager	4	1.43	2.5
	Head Nurse	5	1.79	4.29
Monthly income (RMB)	No	268	95.71	100.00
	1,000–3,000	29	10.36	10.36
	3,000–5,000	54	19.29	29.64
	5,000–8,000	115	41.07	70.71
	8,000–10,000	52	18.57	89.29
	>10,000	30	10.71	100.00
Place of birth	Rural	97	34.64	34.64
	Urban	183	65.36	100.00
Total		280	100.0	100.0

### 3.2 Psychological resilience

#### 3.2.1 Scores of psychological resilience and its dimensions

The mean total psychological resilience score was 49.996 ± 24.854. Among the subscales, tenacity had the highest score (25.836 ± 15.029), followed by strength (16.314 ± 9.459), while optimism had the lowest score (7.846 ± 4.875). Overall, the resilience level of hepatobiliary surgical nurses was lower than the reported national norm.

#### 3.2.2 Factors associated with psychological resilience

One-way ANOVA showed that psychological resilience differed significantly by gender, age, employment type, years of hepatobiliary surgical experience, professional title, and monthly income (all *p* < 0.05). These variables were entered into a multiple linear regression model with psychological resilience as the dependent variable. As shown in Supplementary Table S1, employment type, years of hepatobiliary surgical experience, professional title, and monthly income were identified as significant predictors of psychological resilience (*p* < 0.05), whereas gender and age were not significant in the final model. All variance inflation factor (VIF) values were below 5, indicating no serious multicollinearity. Sensitivity analyses using parsimonious models yielded consistent results for the main predictors.

### 3.3 Occupational stress

#### 3.3.1 Scores of occupational stress and its dimensions

The mean total occupational stress score was 77.146 ± 31.421. Among the five dimensions, patient care-related stress scored the highest (24.050 ± 10.065), followed by management and interpersonal relationships (19.818 ± 8.229), nursing profession and work (15.600 ± 6.373), and workload and time allocation (11.164 ± 4.748). Work environment and equipment/resources scored the lowest (6.514 ± 2.927).

#### 3.3.2 Factors associated with occupational stress

One-way ANOVA revealed significant differences in occupational stress according to gender, age, marital status, employment type, years of hepatobiliary surgical experience, professional title, and monthly income (all *p* < 0.01). These variables were subsequently entered into a multiple linear regression model. As presented in Supplementary Table S2, gender, age, marital status, employment type, and monthly income remained significant predictors of occupational stress in the final model (all *p* < 0.05), whereas years of experience and professional title were not statistically significant. All variance inflation factor (VIF) values were below 5, indicating no serious multicollinearity. Sensitivity analyses using parsimonious models yielded consistent results for the main predictors.

### 3.4 Perceived professional benefits

#### 3.4.1 Scores of perceived professional benefits and its dimensions

The mean total score for perceived professional benefits was 110.314 ± 34.934. Among the subscales, personal growth (26.856 ± 8.936) and positive career perception (26.575 ± 8.621) had relatively higher scores, followed by harmonious nurse–patient relationships (20.154 ± 6.496) and recognition by relatives and friends (19.932 ± 6.440). Sense of team belonging showed the lowest score (16.800 ± 5.457), indicating room for improvement in team cohesion.

#### 3.4.2 Factors associated with perceived professional benefits

Univariate analysis showed that perceived professional benefits differed significantly by gender, age, marital status, employment type, professional title, and monthly income (all *p* < 0.05 or *p* < 0.01). These variables were included in a multiple linear regression model with perceived professional benefits as the dependent variable. As shown in Supplementary Table S3, employment type and monthly income were significant predictors of perceived professional benefits (*p* < 0.05), whereas the other variables were not statistically significant in the final model. All variance inflation factor (VIF) values were below 5, indicating no serious multicollinearity. Sensitivity analyses using parsimonious models yielded consistent results for the main predictors.

### 3.5 Work-related well-being

#### 3.5.1 Scores of work-related well-being and its dimensions

The mean total score of work-related well-being was 78.339 ± 24.638. Among the subscales, work value had the highest score (20.775 ± 6.680), followed by interpersonal relationships (16.650 ± 5.378) and welfare and benefits (16.239 ± 5.360). Management (12.293 ± 4.161) and job characteristics (12.382 ± 4.194) had comparatively lower scores, suggesting that nurses perceived less support from managers and some limitations in job design.

#### 3.5.2 Factors associated with work-related well-being

Univariate analyses indicated that work-related well-being differed significantly by gender, marital status, number of children, employment type, years of hepatobiliary surgical experience, professional title, and monthly income (all *p* < 0.05 or *p* < 0.01). Psychological resilience, occupational stress, and perceived professional benefits were also significantly correlated with work-related well-being. These variables were entered into a multiple linear regression model with work-related well-being as the dependent variable. As shown in Table 2, number of children, employment type, monthly income, psychological resilience, occupational stress, and perceived professional benefits were significant predictors of work-related well-being (all *p* < 0.05). Specifically, psychological resilience and perceived professional benefits were positively associated with work-related well-being, whereas occupational stress had a negative association. All variance inflation factor (VIF) values were below 5, indicating no serious multicollinearity. Sensitivity analyses using parsimonious models yielded consistent results for the main predictors.

**Table 2:** Multiple linear regression analysis of work-related well-being.

**Variable**	**Unstandardized coefficients**	**SE**	**Standardized coefficients**	***t***	***p***	**Collinearity diagnostics**	**Tolerance**
	***B***		**Beta**			**VIF**	
Constant	42.586	15.791	–	2.697	0.007^**^	–	–
Gender	3.438	4.668	0.035	0.737	0.462	1.202	0.832
Marital status	0.075	2.300	0.002	0.033	0.974	1.318	0.759
Number of children	3.685	1.459	0.113	2.525	0.012^*^	1.057	0.946
Employment type	3.283	1.563	0.100	2.100	0.037^*^	1.187	0.842
Years in hepatobiliary surgery	1.423	0.978	0.067	1.454	0.147	1.132	0.883
Professional title	−1.213	1.309	−0.045	−0.927	0.355	1.226	0.816
Monthly income	6.547	1.064	0.294	6.155	0.000^**^	1.205	0.830
Psychological resilience	0.111	0.054	0.112	2.059	0.040^*^	1.556	0.643
Occupational stress	−0.298	0.052	−0.380	−5.744	0.000^**^	2.314	0.432
Perceived professional benefits	0.112	0.037	0.158	3.001	0.003^**^	1.465	0.683

*F* = 25.857, *R*^2^ = 0.490, Δ*R*^2^ = 0.471, ^*^p < 0.05, ^**^p < 0.01.

### 3.6 Correlations among psychological resilience, occupational stress, perceived professional benefits, and work-related well-being

Pearson correlation analysis demonstrated that psychological resilience and perceived professional benefits were positively correlated with work-related well-being (both *r* > 0, *p* < 0.001), whereas occupational stress was negatively correlated with work-related well-being (*r* < 0, *p* < 0.001). In addition, psychological resilience and perceived professional benefits were negatively correlated with occupational stress and positively correlated with each other (Supplementary Table S4). These findings suggest that higher resilience and greater perceived professional benefits are associated with higher levels of work-related well-being and lower levels of occupational stress.

### 3.7 Structural equation modeling

**Figure 1:**
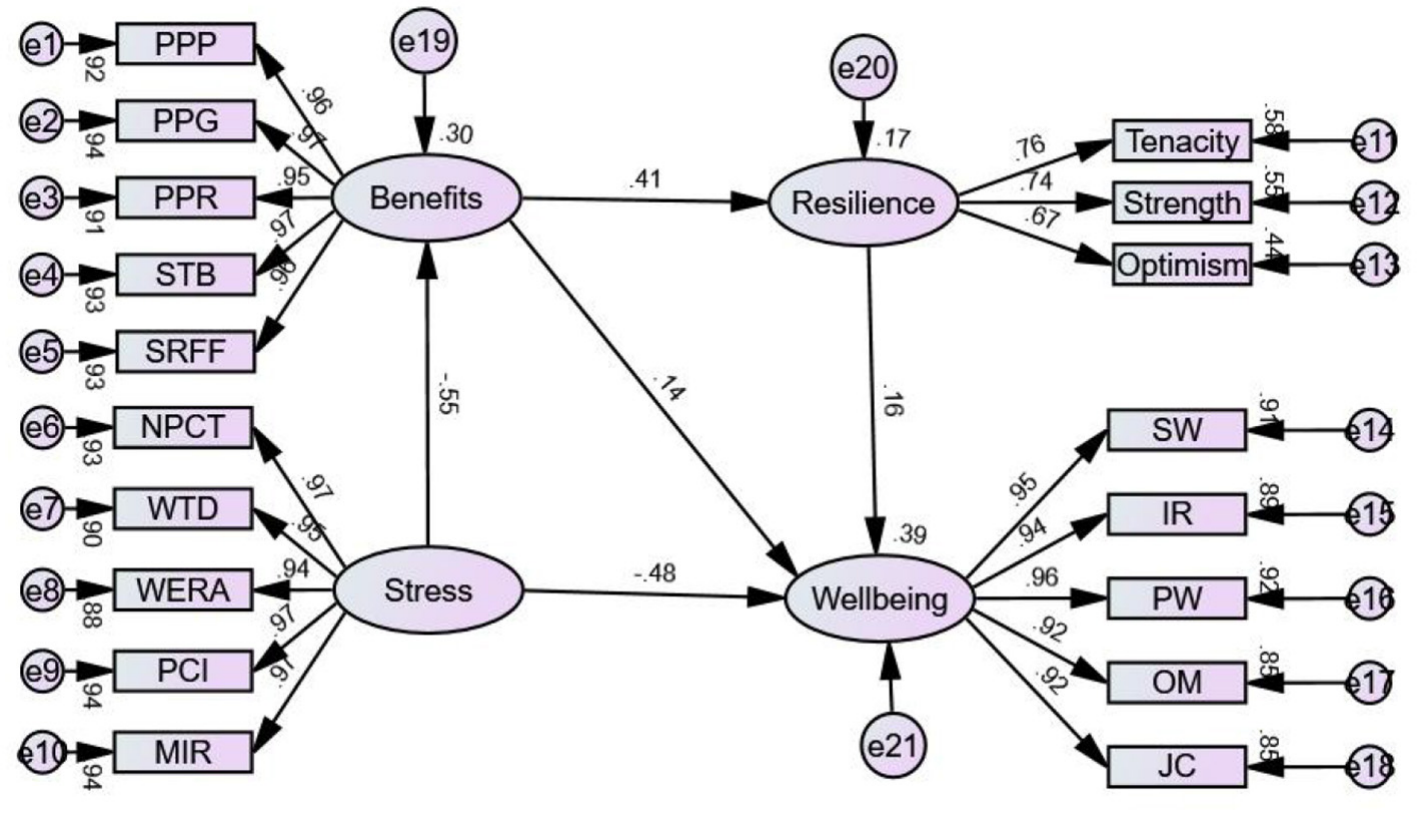
Structural equation model of the associations among occupational stress, perceived professional benefits, psychological resilience, and work-related well-being.

Structural equation modeling was used to further explore the pathways linking psychological resilience, occupational stress, perceived professional benefits, and work-related well-being (Figure 1). The overall model fit was acceptable, with GFI, RMSEA, RMR, CFI, NFI, and NNFI all meeting recommended thresholds (Table 3). All specified paths in the model reached statistical significance (*p* < 0.05). Psychological resilience was directly and positively associated with work-related well-being (standardized path coefficient β = 0.228, *p* < 0.05). Occupational stress was directly and negatively associated with work-related well-being (β = −0.681, *p* < 0.05) and showed an additional indirect association with work-related well-being via perceived professional benefits (indirect association = −0.671 × 0.162 = −0.109). Perceived professional benefits were positively associated with work-related well-being both directly (β = 0.162, *p* < 0.05) and indirectly through psychological resilience (indirect association = 0.332 × 0.228 = 0.076). Overall, the SEM results supported significant associations among psychological resilience, occupational stress, perceived professional benefits, and work-related well-being, with psychological resilience and perceived professional benefits acting as partial mediators in these associations. Detailed standardized path coefficients, standard errors, and hypothesis testing results are provided in Supplementary Table S5.

**Table 3:** Model fit indices of the modified scale.

**Indices**	**Recommended thresholds**	**Value**	**Result**
CMIN/DF	< 3	2.063	Excellent
PGFI	>0.5	0.680	Excellent
NFI	>0.9	0.965	Excellent
RFI	>0.9	0.959	Excellent
IFI	>0.9	0.982	Excellent
TLI	>0.9	0.979	Excellent
CFI	>0.9	0.982	Excellent
PNFI	>0.5	0.820	Excellent
PCFI	>0.5	0.834	Excellent
RMSEA	< 0.10	0.062	Excellent

## 4 Discussion

This study found that hepatobiliary surgical nurses exhibited relatively low levels of psychological resilience compared with the national norm, while their perceived professional benefits were at a moderately low level. These findings suggest that nurses in this high-risk, high-intensity specialty may experience greater challenges related to occupational stress. Hepatobiliary surgery units are characterized by high surgical volumes, complex disease conditions, and frequent postoperative complications, all of which require sustained vigilance and rapid decision-making. Under such circumstances, nurses may experience repeated exposure to critical events and emotional strain, potentially depleting their psychological resources and undermining resilience over time.

Occupational stress in this study mainly originated from patient care, followed by management and interpersonal factors, which is consistent with previous studies conducted among nurses in acute and critical care settings (22–24). Stressors such as fear of clinical errors, responsibility for critically ill patients, and coping with unexpected deterioration or death are particularly salient in hepatobiliary surgery. The relatively lower scores for work environment and equipment suggest that, although material resources may be relatively adequate, the psychological demands of direct patient care remain the primary source of stress (25).

Perceived professional benefits were moderate overall but slightly lower than those reported in some prior studies. Notably, the dimensions of personal growth and positive career perception scored relatively higher, indicating that hepatobiliary nurses are able to gain a sense of competence and professional development through managing diverse and complex clinical conditions. In contrast, the lowest scores were observed for team belongingness, suggesting that deficits in team cohesion and organizational connectedness may weaken nurses' overall sense of benefit from their profession.

With regard to work-related well-being, work value scored the highest among all dimensions, while management and job characteristics scored the lowest. This pattern implies that nurses derive substantial meaning and fulfillment from direct patient care and from perceiving their work as valuable, but may feel less supported by management and less satisfied with aspects of job design such as autonomy, role clarity, and task allocation. These findings highlight the relevance of both organizational and psychological factors in relation to nurses' work-related well-being.

Consistent with the hypotheses, psychological resilience and perceived professional benefits were positively associated with work-related well-being, whereas occupational stress was negatively associated. Nurses with higher resilience tended to report higher levels of work-related well-being, which aligns with previous research suggesting that resilience is linked to better emotional regulation, higher professional efficacy, and lower burnout. As resilient individuals are more capable of adapting to adversity and maintaining goal-directed behavior, they may be better able to preserve a positive outlook and satisfaction with their work despite ongoing challenges.

Perceived professional benefits also showed a significant positive relationship with work-related well-being. When nurses are able to realize self-worth, gain recognition from patients, families, and society, and experience personal and professional growth through their work, they are more likely to develop a strong sense of professional identity and fulfillment. This, in turn, enhances their subjective well-being at work. The present findings are consistent with the view that perceived professional benefits may represent a positive psychological resource associated with motivation and commitment.

By contrast, occupational stress was negatively correlated with work-related well-being, indicating that higher stress levels are associated with diminished positive work experiences. In hepatobiliary surgery, the heavy workload, high acuity, and emotional burden related to complex patient care may result in fatigue, anxiety, and reduced job satisfaction. When stress exceeds nurses' coping capacity and is not effectively buffered by resilience or positive professional experiences, it may erode their sense of well-being and increase the risk of burnout or turnover intention.

Multivariate analysis showed that psychological resilience, perceived professional benefits, occupational stress, number of children, employment type, and monthly income were jointly associated with work-related well-being. Specifically, resilience and professional benefits were positive predictors, whereas occupational stress showed a negative association. These findings suggest that both psychological and contextual factors play important roles in shaping nurses' well-being. Beyond psychological factors, the present findings also highlight the importance of socioeconomic and contractual conditions in shaping nurses' work-related well-being. Employment type and monthly income were independently associated with well-being, suggesting that job stability and adequate financial compensation may contribute to a greater sense of security and satisfaction at work. Nurses employed under more stable contractual arrangements may experience less uncertainty regarding career development and income continuity, which could be reflected in higher levels of well-being. Similarly, sufficient income may alleviate financial stress and support better work–life balance, indirectly relating to more positive work experiences. These results underscore that nurses' well-being is embedded not only in individual psychological resources but also in broader employment and socioeconomic contexts.

Nurses with one child reported higher levels of work-related well-being than those with no children or multiple children, which may reflect a more favorable work–family balance when caregiving responsibilities are moderate rather than minimal or excessive (26, 27). Employment stability and adequate income were also positively associated with well-being, underscoring the importance of job security and financial recognition in enhancing nurses' sense of satisfaction and future orientation.

The structural equation model further illustrated the pattern of associations among these variables. Psychological resilience was directly and positively associated with work-related well-being, suggesting that resilience may represent an important protective psychological resource (28–30). Occupational stress was negatively associated with work-related well-being and also exerted an additional indirect negative effect through perceived professional benefits, suggesting that high stress may weaken nurses' capacity to perceive rewards and meaning from their work (24, 31, 32). At the same time, perceived professional benefits were directly and indirectly associated with work-related well-being, partly through their association with psychological resilience.

Overall, psychological resilience and perceived professional benefits partially mediated the associations between occupational stress and work-related well-being.

These findings support a resource-based perspective, in which psychological resilience and perceived professional benefits form an internal reservoir that allows nurses to cope more effectively with occupational stress and maintain a positive evaluation of their work. For hepatobiliary surgical nurses, who are exposed to continuous clinical challenges, strengthening these psychological resources may be particularly important for sustaining long-term well-being and professional engagement. Taken together, these findings suggest that work-related well-being among hepatobiliary surgical nurses is associated with an interplay between individual psychological resources and structural socioeconomic and contractual conditions.

Although causal inferences cannot be drawn from this cross-sectional study, the observed associations may have practical implications for nursing management. The present study provides several practical implications for nursing managers in hepatobiliary surgery and similar high-intensity units. First, interventions aimed at reducing excessive occupational stress remain essential. Optimizing staffing and shift schedules may be associated with more favorable working conditions, which in turn may support nurses' work-related well-being. Timely debriefing and emotional support following critical incidents may also help nurses process stressful experiences more effectively. Second, strategies to enhance psychological resilience should be incorporated into continuing education and professional development programs. Evidence-based resilience interventions, such as mindfulness training, cognitive–behavioral techniques, and stress management workshops, may help nurses develop more adaptive coping styles and emotional regulation skills. Flexible work arrangements and supportive supervision may further reinforce resilience by providing a more secure and predictable work environment. Third, nursing managers should actively foster nurses' perceived professional benefits. This may include creating opportunities for career development, encouraging participation in clinical decision-making, recognizing outstanding performance, and enhancing team communication and cohesion. For nurses with heavier family responsibilities or less stable employment status, targeted support—such as flexible scheduling, fair promotion policies, and transparent compensation schemes—may help reduce perceived inequities and promote a stronger sense of belonging.

## 5 Limitations

This study has several limitations. First, the cross-sectional design precludes causal inferences regarding the relationships among psychological resilience, occupational stress, perceived professional benefits, and work-related well-being. Longitudinal or intervention studies are needed to further clarify causal pathways. Second, convenience sampling from hospitals within a single province may limit the generalizability of the findings to other regions or healthcare settings. Future research should include multi-center samples from different geographic and institutional contexts. Third, all data were collected through self-report questionnaires, which may be subject to recall bias and social desirability effects. Incorporating objective indicators (e.g., turnover, sickness absence) and qualitative methods could provide a more comprehensive understanding of nurses' well-being. In addition, although all measurement instruments used in this study were established Chinese versions with documented reliability and validity, confirmatory factor analysis was not conducted to re-examine structural validity in the current sample. Future studies may further assess the factorial structure of these instruments using independent samples.

## 6 Conclusions

Despite these limitations, the present study highlights the crucial roles of psychological resilience and perceived professional benefits in buffering occupational stress and promoting work-related well-being among hepatobiliary surgical nurses. These findings suggest that perceived professional benefits may function as an important positive psychological resource associated with work-related well-being.

## Data Availability

The original contributions presented in the study are included in the article/Supplementary material, further inquiries can be directed to the corresponding author.

## References

[1] Gündüz ES Yildirim N Akatin Y Gündogdu NA. Relationship between nurses' resilience and quality of professional life. Int Nurs Rev. (2024) 71:1023–31. doi: 10.1111/inr.12960 38511869

[2] Alonazi O Alshowkan A Shdaifat E. The relationship between psychological resilience and professional quality of life among mental health nurses: a cross-sectional study. BMC Nurs. (2023) 22:184. doi: 10.1186/s12912-023-01346-1 37248491 PMC10228012

[3] Foster K Roche M Giandinoto JA Furness T. Workplace stressors, psychological well-being, resilience, and caring behaviours of mental health nurses: a descriptive correlational study. Int J Ment Health Nurs. (2020) 29:56–68. doi: 10.1111/inm.12610 31127973

[4] Li LZ Yang P Singer SJ Pfeffer J Mathur MB Shanafelt T. Nurse burnout and patient safety, satisfaction, and quality of care: a systematic review and meta-analysis. JAMA Netw Open. (2024) 7:e2443059. doi: 10.1001/jamanetworkopen.2024.43059 39499515 PMC11539016

[5] Giabicani M Froissant PA Weiss E. Critical care challenges in hepatobiliary and pancreatic surgery. Curr Opin Crit Care. (2025) 31:750–6. doi: 10.1097/MCC.0000000000001332 41198641

[6] Shi L Zhang J Xiao S Lin H Zhao C Zhao S . Impact of occupational exposure on job satisfaction and overall happiness among Chinese physicians and nurses: a cross-sectional study. J Nurs Manag. (2022) 30:2062–73. doi: 10.1111/jonm.13663 35506574

[7] Cooper AL Brown JA Rees CS Leslie GD. Nurse resilience: a concept analysis. Int J Ment Health Nurs. (2020) 29:553–75. doi: 10.1111/inm.12721 32227411

[8] Troy AS Willroth EC Shallcross AJ Giuliani NR Gross JJ Mauss IB. Psychological resilience: an affect-regulation framework. Annu Rev Psychol. (2023) 74:547–76. doi: 10.1146/annurev-psych-020122-041854 36103999 PMC12009612

[9] Alkhawaldeh JMA Soh KL Mukhtar FBM Ooi CP. Effectiveness of stress management interventional programme on occupational stress for nurses: a systematic review. J Nurs Manag. (2020) 28:209–20. doi: 10.1111/jonm.12938 31887233

[10] Green AA Kinchen EV. The effects of mindfulness meditation on stress and burnout in nurses. J Holist Nurs. (2021) 39:356–68. doi: 10.1177/08980101211015818 33998935

[11] Talebiazar N Anzali BC Abbasi M Azizi N Goli R Faraji N . Does mindfulness-based stress reduction training have an impact on the occupational burnout and stress experienced by nurses? A randomized controlled trial. Int Arch Occup Environ Health. (2025) 98:1–11. doi: 10.1007/s00420-024-02078-8 39601884

[12] Chen C Sun X Zhang Y Liu Z Jiao M Hu Y. Mediating effect of perceived professional benefit on the relationship between spiritual health and spiritual care competence among new nurses: a cross-sectional study. J Nurs Manag. (2025) 2025:8832454. doi: 10.1155/jonm/8832454 40351854 PMC12064319

[13] Liu S Duan X Han P Shao H Jiang J Zeng L. Occupational benefit perception of acute and critical care nurses: a qualitative meta-synthesis. Front Public Health. (2022) 10:976146. doi: 10.3389/fpubh.2022.976146 36249239 PMC9561925

[14] Castillo-González A Velando-Soriano A De La Fuente-Solana EI Martos-Cabrera BM Membrive-Jiménez MJ Lucía RB . Relation and effect of resilience on burnout in nurses: a literature review and meta-analysis. Int Nurs Rev. (2024) 71:160–7. doi: 10.1111/inr.12838 37000679

[15] Zhai X Ren LN Liu Y Liu CJ Su XG Feng BE. Resilience training for nurses: a meta-analysis. J Hosp Palliat Nurs. (2021) 23:544–50. doi: 10.1097/NJH.0000000000000791 34313624

[16] Chen C Meier ST. Burnout and depression in nurses: a systematic review and meta-analysis. Int J Nurs Stud. (2021) 124:104099. doi: 10.1016/j.ijnurstu.2021.104099 34715576

[17] Jarden RJ Jarden A Weiland TJ Taylor G Bujalka H Brockenshire N . New graduate nurse wellbeing, work wellbeing and mental health: a quantitative systematic review. Int J Nurs Stud. (2021) 121:103997. doi: 10.1016/j.ijnurstu.2021.103997 34218048

[18] Kunzler AM Helmreich I Chmitorz A König J Binder H Wessa M . Psychological interventions to foster resilience in healthcare professionals. Cochrane Database Syst Rev. (2020) 7:Cd012527. doi: 10.1002/14651858.CD012527.pub2 32627860 PMC8121081

[19] Sun C Jiang H Yao Q Wang X Wen X Liu H. Latent profile analysis of nurses' perceived professional benefits in China: a cross-sectional study. BMJ Open. (2023) 13:e078051. doi: 10.1136/bmjopen-2023-078051 37918934 PMC10626806

[20] Demerouti E Bakker AB Nachreiner F Schaufeli WB. The job demands-resources model of burnout. J Appl Psychol. (2001) 86:499–512. doi: 10.1037/0021-9010.86.3.499 11419809

[21] Bakker AB Demerouti E. Job demands-resources theory: taking stock and looking forward. J Occup Health Psychol. (2017) 22:273–85. doi: 10.1037/ocp0000056 27732008

[22] Jun J Ojemeni MM Kalamani R Tong J Crecelius ML. Relationship between nurse burnout, patient and organizational outcomes: systematic review. Int J Nurs Stud. (2021) 119:103933. doi: 10.1016/j.ijnurstu.2021.103933 33901940

[23] Nowrouzi B Nguyen C Casole J Nowrouzi-Kia B. Occupational stress: a comprehensive review of the top 50 annual and lifetime cited articles. Workplace Health Saf. (2017) 65:197–209. doi: 10.1177/2165079916666300 27758938

[24] Okuhara M Sato K Kodama Y. The nurses' occupational stress components and outcomes, findings from an integrative review. Nursing open. (2021) 8:2153–74. doi: 10.1002/nop2.780 33635606 PMC8363363

[25] Wheeler HH. Nurse occupational stress research 3: a model of stress for research. Br J Nurs. (1997) 6:944–9. doi: 10.12968/bjon.1997.6.16.944 9362626

[26] Granrud MD Sandsdalen T Anderzén-Carlsson A Steffenak AKM. Public health nurses' experiences working with children who are next of kin: a qualitative study. BMC Health Serv Res. (2022) 22:1427. doi: 10.1186/s12913-022-08841-2 36443847 PMC9703408

[27] Ha EH. Attitudes toward child rearing in female clinical nurses working in three shifts. Nurs Health Sci. (2016) 18:416–24. doi: 10.1111/nhs.12284 27098460

[28] Imran A Tariq S Kapczinski F de Azevedo Cardoso T. Psychological resilience and mood disorders: a systematic review and meta-analysis. Trends Psychiatry Psychother. (2024) 46:e20220524. doi: 10.47626/2237-6089-2022-0524 36215270 PMC11332678

[29] Sisto A Vicinanza F Campanozzi LL Ricci G Tartaglini D Tambone V. Towards a transversal definition of psychological resilience: a literature review. Medicina. (2019) 55:745. doi: 10.3390/medicina55110745 31744109 PMC6915594

[30] Tay PKC Lim KK. Psychological resilience as an emergent characteristic for well-being: a pragmatic view. Gerontology. (2020) 66:476–83. doi: 10.1159/000509210 32784301

[31] Singh C Cross W Munro I Jackson D. Occupational stress facing nurse academics-a mixed-methods systematic review. J Clin Nurs. (2020) 29:720–35. doi: 10.1111/jocn.15150 31856356

[32] Zhang M Murphy B Cabanilla A Yidi C. Physical relaxation for occupational stress in healthcare workers: a systematic review and network meta-analysis of randomized controlled trials. J Occup Health. (2021) 63:e12243. doi: 10.1002/1348-9585.12243 34235817 PMC8263904

