# Supplementary material for: Work-related well-being among hepatobiliary surgical nurses: a structural equation modeling study
Source: Front Public Health. 2026 Feb 12;14:1772238. doi: 10.3389/fpubh.2026.1772238 (PMC12935591; doi:10.3389/fpubh.2026.1772238)
Supplement: Supplementary file 1 [file Data_Sheet_1.docx]

Table S1. Factors associated with psychological resilience.

| **Variable** | **Unstandardized coefficients** |  | **Standardized coefficients** | **t** | **p** |
| --- | --- | --- | --- | --- | --- |
|  | **B** | **SE** | **Beta** |  |  |
| Constant | 1.669 | 12.725 | – | 0.131 | 0.896 |
| Gender | 7.267 | 5.382 | 0.074 | 1.350 | 0.178 |
| Age | 1.356 | 1.459 | 0.051 | 0.930 | 0.353 |
| Employment type | −3.997 | 1.827 | −0.120 | −2.187 | 0.030* |
| Years in hepatobiliary surgery | 2.488 | 1.171 | 0.117 | 2.125 | 0.034* |
| Professional title | 8.239 | 1.519 | 0.301 | 5.424 | 0.000** |
| Monthly income | 4.592 | 1.208 | 0.205 | 3.800 | 0.000** |

F = 13.948, R² = 0.235, ΔR² = 0.218, p < 0.01

Table S2. Factors associated with occupational stress.

| **Variable** | **Unstandardized coefficients** |  | **Standardized coefficients** | **t** | **p** | **Collinearity diagnostics** |  |
| --- | --- | --- | --- | --- | --- | --- | --- |
|  | **B** | **SE** | **Beta** |  |  | **VIF** | **Tolerance** |
| Constant | 197.421 | 14.332 | – | 13.775 | 0.000** | – | – |
| Gender | −26.763 | 6.007 | −0.215 | −4.456 | 0.000** | 1.105 | 0.905 |
| Age | −6.635 | 1.643 | −0.198 | −4.039 | 0.000** | 1.140 | 0.877 |
| Marital status | −13.811 | 2.971 | −0.235 | −4.649 | 0.000** | 1.220 | 0.820 |
| Employment type | 6.684 | 2.036 | 0.159 | 3.282 | 0.001** | 1.118 | 0.894 |
| Years in hepatobiliary surgery | −2.316 | 1.294 | −0.086 | −1.790 | 0.075 | 1.100 | 0.909 |
| Professional title | −3.228 | 1.662 | −0.093 | −1.942 | 0.053 | 1.097 | 0.911 |
| Monthly income | −8.040 | 1.328 | −0.283 | −6.052 | 0.000** | 1.044 | 0.958 |

F = 29.194, R² = 0.429, ΔR² = 0.414, p < 0.01

Table S3. Factors associated with perceived professional benefits.

| **Variable** | **Unstandardized coefficients** |  | **Standardized coefficients** | **t** | **p** | **Collinearity diagnostics** |  |
| --- | --- | --- | --- | --- | --- | --- | --- |
|  | **B** | **SE** | **Beta** |  |  | **VIF** | **Tolerance** |
| Constant | 70.120 | 19.331 | – | 3.627 | 0.000** | – | – |
| Gender | 5.836 | 8.237 | 0.042 | 0.709 | 0.479 | 1.094 | 0.914 |
| Age | 0.161 | 2.263 | 0.004 | 0.071 | 0.943 | 1.139 | 0.878 |
| Marital status | 7.251 | 4.052 | 0.111 | 1.789 | 0.075 | 1.195 | 0.837 |
| Employment type | −7.186 | 2.800 | −0.154 | −2.566 | 0.011* | 1.114 | 0.898 |
| Professional title | 4.245 | 2.227 | 0.110 | 1.906 | 0.058 | 1.036 | 0.965 |
| Monthly income | 6.395 | 1.830 | 0.203 | 3.495 | 0.001** | 1.043 | 0.959 |

F = 6.169, R² = 0.119, ΔR² = 0.100, p < 0.01

Table S4. Correlations among psychological resilience, occupational stress, perceived professional benefits, and work-related well-being.

|  | 1 | 2 3 | 4 | 5 | 6 | 7 | 8 | 9 | 10 | 11 | 12 | 13 | 14 | 15 | 16 | 17 |
| --- | --- | --- | --- | --- | --- | --- | --- | --- | --- | --- | --- | --- | --- | --- | --- | --- |
| Work-related well-being | 1 |  |  |  |  |  |  |  |  |  |  |  |  |  |  |  |
| Psychological Resilience | 0.403  ** | 0.917 0.816  ** ** | 0.689  ** | 1 |  |  |  |  |  |  |  |  |  |  |  |  |
| Work Stress | -0.602  ** | -0.426-0.398  ** ** | -0.384  ** | -0.484  ** | 0.972  ** | 0.957*  * | 0.943  ** | 0.982  ** | 0.978  ** | 1 |  |  |  |  |  |  |
| Sense of Professional Benefit | 0.443  ** | 0.309 0.290  ** ** | 0.273  ** | 0.351  ** | -0.520  ** | -0.482  ** | -0.511  ** | -0.524  ** | -0.556  ** | -0.539  ** | 0.977  ** | 0.978  ** | 0.963  ** | 0.967  ** | 0.970  ** | 1 |

Table S5. Direct Path Coefficients and Hypothesis Testing Results.

| Direct path | | | Estimate | S.E. | C.R. | P | Label | Result |
| --- | --- | --- | --- | --- | --- | --- | --- | --- |
| Benefits | <--- | Stress | -0.55 | 0.065 | -10.49 | *** | H1a | Supported |
| Resilience | <--- | Benefits | 0.408 | 0.055 | 5.947 | *** | H2a | Supported |
| Wellbeing | <--- | Resilience | 0.157 | 0.096 | 2.347 | 0.019 | H3 | Supported |
| Wellbeing | <--- | Benefits | 0.137 | 0.07 | 2.271 | 0.023 | H2b | Supported |
| Wellbeing | <--- | Stress | -0.475 | 0.091 | -7.456 | *** | H1b | Supported |
